# Differing impacts of cardiac implantable electronic device leads on tricuspid regurgitation

**DOI:** 10.1002/joa3.70133

**Published:** 2025-07-07

**Authors:** Sophie A. Leon, Melissa Austin, Nayeem Nasher, Daler Rahimov, Faizaan Siddique, Chitra Parikh, Danial Ahmad, Vakhtang Tchantchaleishvili, Behzad B. Pavri

**Affiliations:** ^1^ Department of Cardiac Surgery Thomas Jefferson University Hospital Philadelphia Pennsylvania USA; ^2^ Division of Cardiology, Department of Medicine Thomas Jefferson University Hospital Philadelphia Pennsylvania USA

**Keywords:** defibrillator, his bundle pacemaker, pacemaker, tricuspid regurgitation

## Abstract

**Background:**

Placement of cardiac implantable electronic devices (CIED) with leads that traverse the tricuspid valve is thought to contribute to tricuspid regurgitation (TR). However, there are relatively limited data comparing the impact of different CIED lead types on the incidence and progression of TR. This study sought to quantify the change in TR severity following implantation of CIEDs with different lead types.

**Methods:**

Patient data were collected on individuals with implantable cardioverter defibrillators (ICD), right ventricular‐paced pacemakers (RV‐PM), and His bundle‐paced pacemakers (His‐PM) placed by a single provider at a single institution between 2016 and 2019. Patients with extravascular CIED placement or with existing devices undergoing upgrade procedures were excluded. Severity of TR after CIED implantation was compared to baseline TR.

**Results:**

A total of 97 patients receiving CIEDs were analyzed, including 63 with RV‐PMs, 23 with ICDs, and 11 with His‐PMs. Median patient age was 72 [interquartile range (IQR) 63–81] years, and 44% of patients were female. Echocardiograms were obtained a median of 20 [4–91] days before CIED implantation and 31 [17.9–43.0] months following implantation. Baseline TR grade was comparable between groups (*p* = 0.65). TR severity significantly worsened after ICD implantation (*p* = 0.035), RV‐PM implantation trended toward worsening TR severity (*p* = 0.099), and no statistically significant difference was observed after His‐PM implantation (*p* = 0.68).

**Conclusion:**

The effect of CIED leads on TR represents a spectrum related to the type of lead traversing the tricuspid valve.

## Graphical abstract

This study evaluates the impact of different CIED lead types (ICD, RV‐PM, His‐PM) on the progression of tricuspid regurgitation (TR). The effect of CIED leads on TR grade represents a spectrum related to the type of lead traversing the tricuspid valve.

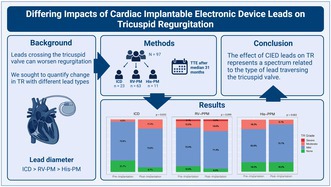

## 1 INTRODUCTION

The tricuspid valve has long been forgotten in the cardiovascular world.^1^ For instance, the prevalence of moderate‐to‐severe TR was reported to affect 1.6 million patients in the United States, of which less than 8000 have had any surgical intervention.^2^ For decades, there was a longstanding notion that addressing left‐sided pathologies should resolve any concomitant TR but that has not been the case.^1^ The repercussions of undiagnosed or untreated TR have increasingly grown in clinical importance as it is associated with significant morbidity and mortality.^3^ Placement of a cardiac implantable electronic device (CIED), while often necessary, is thought to induce or worsen TR directly through mechanical interference including iatrogenic injury during initial device implantation or subsequent lead extraction as well as mechanical impingement of the leads on the valvular apparatus. CIEDs may also worsen TR secondarily through electromechanical dyssynchrony along with the ensuing structural changes in the heart observed with disease progression.^4^

TR secondary to CIED placement introduces further challenges to a population already afflicted with conduction abnormalities. The incidence of new onset or worsening TR following CIED placement varies according to the literature and is reported to be as high as 45% depending on the study population.^5^ Depending on the patient's presentation, management of TR may necessitate removal and replacement of the leads, exchanging for an updated device configuration, and in severe cases, surgical repair or replacement.^6^

Several reports have described TR following CIED placement; however, there is a relative paucity of data measuring the degree of TR as it relates to device type. Thus, we sought to quantify the change in severity of TR following implantation of different CIEDs according to types of leads utilized.

## 2 METHODS

### 2.1 Study design

We identified all patients who underwent placement of CIEDs by a single provider at our institution from January 2016 to October 2019. We conducted a retrospective chart review in which patient data were extracted and de‐identified. Extracted data included baseline demographic information, indications for CIED placement, device type, follow‐up time, and echocardiographic data pre‐ and postdevice placement. The study design was approved by the Institutional Review Board (IRB) under protocol number 21E.629, with a waiver of informed consent.

Echocardiographic data were collected from reports that met the following criteria: baseline report <365 days prior to device implantation, and follow‐up report >90 days postdevice implantation. Echocardiogram reports were reviewed to classify TR as mild, moderate, or severe. TR reported as mild–moderate was classified as moderate, and TR reported as moderate–severe was classified as severe. Left ventricular ejection fraction (LVEF) was reported using the Simpson method, and EF ranges were reported as their average value. Right ventricular (RV) size was classified as normal vs. dilated, and RV function was classified as normal vs. reduced, as denoted in the echocardiogram report.

### 2.2 Inclusion and exclusion criteria

All adult patients who underwent de novo transvenous CIED placement were included in our study. The devices included ICDs, right ventricular‐paced pacemakers (RV‐PM), and His bundle‐paced pacemakers (His‐PM). RV‐PM comprised all pacemakers with at least one lead located in the RV, which included single‐chamber ventricular and dual‐chamber pacemakers. ICD leads were placed in the right ventricular apex and RV‐PM leads were placed mid‐septum. Patients who underwent subcutaneous ICD placement, biventricular pacemaker placement, atrial‐only pacemakers, leadless pacemaker placement, patients without pre‐ and postimplant echocardiograms, and patients with prior CIEDs who underwent upgrade procedures were excluded.

### 2.3 Statistical analysis

Demographic information and clinical data were analyzed. Categorical variables were reported as percentages, and continuous variables were reported as medians with interquartile ranges (IQR). Wilcoxon rank‐sum test was used to compare characteristics between each group. The severity of TR, which was the primary outcome of our analysis, was compared before and after CIED implantation using a one‐sided Wilcoxon‐signed rank test, with no change in TR as the null hypothesis and the progression of TR as the alternative hypothesis. Given the small sample size, we opted for the one‐sided test over the two‐sided version (Table S1), as one‐sided tests are generally more powerful when the alternative hypothesis is correctly specified. The direction of the alternative hypothesis was informed by conducting ordinal logistic regression, with categories of TR severity predicted over the time following device implantation (Figures [Link], [Link], [Link]). For each group, odds ratios were calculated (Table S2). The results suggested a possible (albeit nonsignificant) worsening of TR, with the probability of more severe forms of TR steadily rising as the time following implantation increased. This supported our choice of a one‐sided test under the assumption that any change in regurgitation severity post‐implantation would reflect worsening TR.

For other variables, no pretest assumptions were held and, therefore, a two‐sided Wilcoxon‐signed rank test was applied.

Potential confounders, including RV pacing rate, post‐CIED LVEF, and pacing mode (DDD vs. VVI), were analyzed to assess their relationship with TR grades. Correlation analyses were conducted to evaluate the relationships between RV pacing rate and TR grade as well as between post‐CIED LVEF and TR grade.

*p*‐values of <0.05 were considered significant. Statistical analysis was performed using R software, version 4.3.1 (R Foundation for Statistical Computing, Vienna, Austria).

## 3 RESULTS

### 3.1 Baseline characteristics

There were 97 patients who underwent transvenous CIED placement by a single provider between 2016 and 2019 who met inclusion criteria. Median age was 72 [IQR 63–81] years, and 44% were female. Of the CIEDs analyzed, RV‐PMs were the most frequently implanted device (66%), followed by ICDs (28%) and His‐PMs (12%). See Table 1 for further details.

**TABLE 1:** Patient demographics.

	ICD (*n* = 23)	RV pacing (*n* = 63)	His bundle pacing (*n* = 11)	Total (*n* = 97)
Age (years), median [IQR]	65.1 [57.7**–**70.5]	73.3 [65.0**–**83.4]	77 [71.6**–**80.3]	71.5 [62.6**–**81.2]
Sex				
Female, *n* (%)	4 (17.4)	35 (55.6)	4 (36.4)	43 (44.3)
Race, *n* (%)				
White	9 (39.1)	42 (66.7)	11 (100.0)	62 (63.9)
Black	8 (34.8)	15 (23.8)	0 (0.0)	23 (23.7)
Asian	2 (8.7)	1 (1.6)	0 (0.0)	3 (3.1)
Hispanic	2 (8.7)	4 (6.3)	0 (0.0)	6 (6.2)
Other	2 (8.7)	1 (1.6)	0 (0.0)	3 (3.1)

Abbreviations: ICD, implantable cardioverter defibrillator; RV, right ventricular.

### 3.2 Indications for implantation

In patients who underwent pacemaker placement, the most common indication for RV‐PM placement was sick sinus syndrome (59%) and for His‐PM placement was Mobitz type II heart block (27%). For patients undergoing ICD placement, the most common indications were non‐ischemic dilated cardiomyopathy (39%), followed by ischemic cardiomyopathy (22%). See Table 2 for further details.

**TABLE 2:** Cardiac implantable electronic device indication.

Indications	RV pacing (*n* = 63)	His bundle pacing (*n* = 11)	ICD (*n* = 23)
Sick sinus syndrome, *n* (%)	37 (58.73)	2 (18.18)	0 (0.00)
Complete heart block, *n* (%)	13 (20.63)	1 (9.09)	0 (0.00)
Mobitz II heart block, *n* (%)	4 (6.35)	3 (27.27)	0 (0.00)
Bifascicular block, *n* (%)	3 (4.76)	0 (0.00)	0 (0.00)
Carotid hypersensitivity, *n* (%)	2 (3.17)	0 (0.00)	0 (0.00)
High‐grade atrioventricular block, *n* (%)	1 (1.59)	0 (0.00)	0 (0.00)
Stokes‐Adams attack, *n* (%)	1 (1.59)	2 (18.18)	0 (0.00)
Chronic atrial fibrillation (ablate and pace), *n* (%)	0 (0.00)	2 (18.18)	0 (0.00)
Implantable cardiac monitor, *n* (%)	1 (1.59)	0 (0.00)	5 (21.74)
DIlated cardiomyopathy, *n* (%)	0 (0.00)	1 (9.09)	9 (39.13)
Hypertrophic cardiomyopathy, *n* (%)	0 (0.00)	0 (0.00)	1 (4.35)
Cardiac arrest from Ventricular fibrillation or sustained ventricular tachycardia, *n* (%)	0 (0.00)	0 (0.00)	3 (13.04)
Sustained ventricular tachycardia with syncope during EPS, *n* (%)	0 (0.00)	0 (0.00)	1 (4.35)
Sustained ventricular tachycardia with structural heart disease, *n* (%)	0 (0.00)	0 (0.00)	1 (4.35)
Symptomatic non‐sustained ventricular tachycardia, *n* (%)	0 (0.00)	0 (0.00)	1 (4.35)
Arrhythmogenic right ventricular dysplasia, *n* (%)	0 (0.00)	0 (0.00)	1 (4.35)
Other, *n* (%)	1 (1.59)	0 (0.00)	1 (4.35)

### 3.3 Echocardiography findings

**FIGURE 1:**
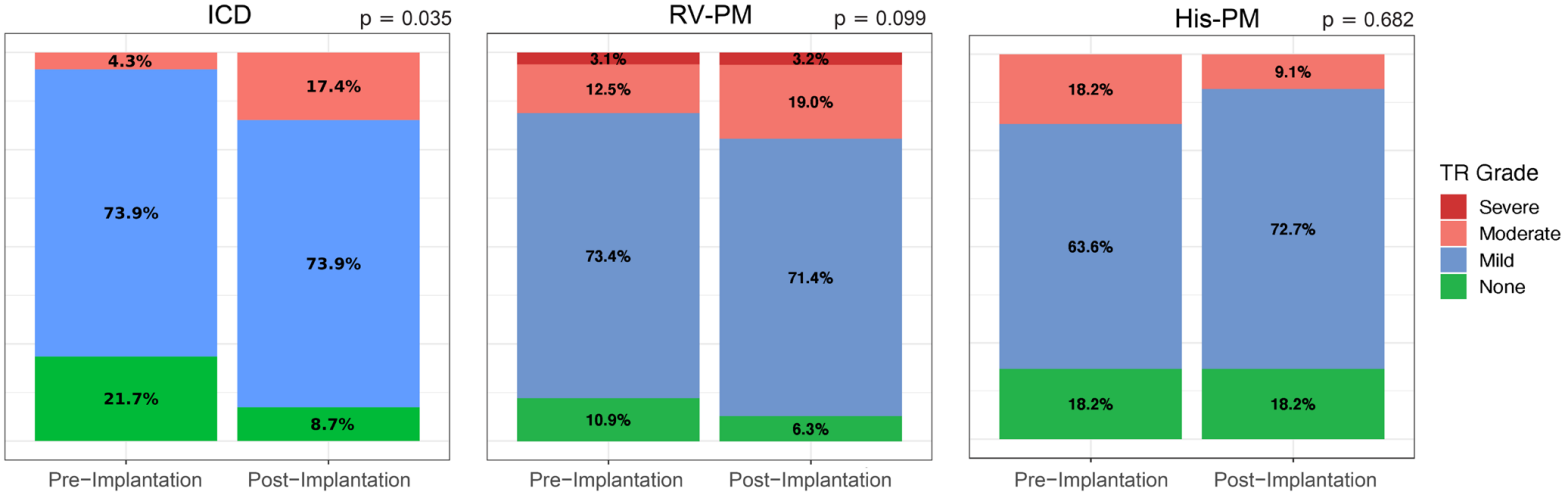
Grade of TR pre‐ and post‐CIED implantation according to CIED type. CIED, cardiac implantable electronic device.

Across all devices, the most common grade of TR preimplantation was mild, comprising 72% of patients at a median time of 20 [4–91] days prior to implantation. The most common TR grade postimplantation was moderate, comprising 72% of patients at a median time of 31 [17.9–43.0] months follow‐up. According to the type of device implanted, ICD placement was significantly associated with worsening of TR severity (*p* = 0.04), whereas RV‐PM placement demonstrated a trend toward worsening severity of TR without statistical significance (*p* = 0.10). There were no significant differences in TR following His‐PM placement (*p* = 0.68). See Table 3 and Figure 1 for further details.

**TABLE 3:** Degree of tricuspid regurgitation reported by echocardiogram.

	Implantable Defibrillator (*n* = 23)	RV pacing (*n* = 63)	His bundle pacing (*n* = 11)	Total (*n* = 97)	*p*‐value
Time from pre‐implant echocardiogram to implant (days), median [IQR]	26 [6.5**–**60.5]	19 [2**–**96]	20 [2.5**–**129.5]	20 [4**–**91]	0.87
Time from implant to last echocardiogram (months), median [IQR]	34 [11.7**–**44.7]	25.4 [18.5**–**42.2]	35.4 [21.8**–**47.6]	30.9 [17.8**–**43.0]	0.49
Tricuspid regurgitation, *n* (%)					
Preimplant					
None	5 (21.7)	7 (11.1)	2 (18.2)	14 (14.4)	
Mild	17 (73.9)	46 (73.0)	7 (63.6)	70 (72.2)	
Moderate	1 (4.3)	8 (12.7)	2 (18.2)	11 (11.3)	
Severe	0 (0.0)	2 (3.2)	0 (0.0)	2 (2.1)	0.65
Postimplant					
None	2 (8.7)	4 (6.3)	2 (18.2)	8 (8.2)	
Mild	4 (17.4)	12 (19.0)	1 (9.1)	17 (17.5)	
Moderate	17 (73.9)	45 (71.4)	8 (72.7)	70 (72.2)	
Severe	0 (0.0)	2 (3.2)	0 (0.0)	2 (2.1)	0.79
*p*‐value by group					
One‐sided, pre < post	0.04	0.10	0.68		

Abbreviations: ICD, implantable cardioverter defibrillator; RV, right ventricular.

Preimplantation RV dilation was higher in the ICD group (26%) than in His‐PM (11%) and RV‐PM (8%) (*p* = 0.040). Patients in the ICD group also demonstrated a significantly higher rate of reduced postimplantation RV function (41%), compared to RV‐PM (5%) and His‐PM (0%) (*p* < 0.001). Across all devices, patients who underwent ICD placement had significantly lower postimplantation left ventricular ejection fraction (LVEF) at 35% [28–44] compared to His‐PM with 59% [46–60] or RV‐PM with 60% [55–65] (*p* < 0.001).

Within the ICD group, when comparing pre‐ vs. postimplantation there were no differences in the observed rates of RV dilation (26% vs. 27%, *p* = 1.0) or reduced RV function (35% vs. 41%, *p* = 0.766). See Table 4 for further details.

**TABLE 4:** Echocardiographic data.

	ICD (*n* = 23)	RV pacing (*n* = 64)	His bundle pacing (*n* = 11)	Total (*n* = 97)	*p*‐value
LVEF (%), median [IQR]					
Preimplant	30 [20**–**42]	61.8 [57.9**–**65.0]	60 [47.5**–**61.9]	60 [40**–**65]	<0.001
Postimplant	35 [27.5**–**44]	60 [55**–**65]	58.8 [46.2**–**60.0]	57.5 [42.0**–**62.5]	<0.001
*p*‐value by group	0.31	0.12	0.91	0.58	
RV size, *n* (%)					
Preimplant					
Normal	17 (73.9)	59 (92.2)	8 (88.9)	84 (88.4)	
Dilated	6 (26.1)	5 (7.8)	1 (11.1)	11 (11.6)	0.04
Postimplant					
Normal	16 (72.7)	56 (90.3)	8 (72.7)	79 (84.0)	
Dilated	6 (27.3)	6 (9.7)	3 (27.3)	15 (16.0)	0.09
*p*‐value by group	1.0	0.48	0.35	0.27	
RV function, *n* (%)					
Preimplant					
Normal	15 (65.2)	61 (96.8)	7 (77.8)	83 (87.4)	
Reduced	8 (34.8)	2 (3.2)	2 (22.2)	12 (12.6)	<0.001
Postimplant					
Normal	13 (59.1)	59 (95.2)	11 (100.0)	83 (87.4)	
Reduced	9 (40.9)	3 (4.8)	0 (0.0)	12 (12.6)	<0.001
*p*‐value by group	0.77	0.77	0.35	0.81	
TAPSE (cm), median [IQR]					
Preimplant	1.9 [1.6**–**2.3]	2 [1.8**–**2.4]	2.6 [2.3**–**2.8]	2 [1.8**–**2.4]	0.06
Postimplant	2 [1.9**–**2.1]	1.9 [1.7**–**2.2]	2 [2.0**–**2.3]	2 [1.8**–**2.2]	0.45
*p*‐value by group	0.24	0.28	0.75	0.13	
PA pressure (mmHg), median [IQR]					
Preimplant	32 [27**–**40]	34 [29.0**–**40.5]	36 [34.0**–**43.5]	34.5 [28.2**–**40.0]	0.43
Postimplant	31 [24**–**49]	32 [28.0**–**42.5]	35 [33**–**44]	33 [28**–**45]	0.45
*p*‐value by group	0.73	1.0	0.87	0.86	
RA pressure (mmHg), median [IQR]					
Preimplant	5 [3.0**–**9.5]	5 [3.0**–**9.5]	9 [7.2**–**11.2]	5 [3**–**10]	0.25
Postimplant	3 [3.0**–**4.2]	3 [3**–**8]	8 [3.0**–**11.2]	3 [3**–**8]	0.29
*p*‐value by group	0.21	0.63	0.89	0.42	
Maximum TR velocity (cm/s), median [IQR]					
Preimplant	245.5 [227.2**–**250.8]	261 [233**–**290]	258 [257.0**–**289.8]	253 [233.5**–**287.5]	0.31
Postimplant	244.5 [218.5**–**305.2]	270 [246.8**–**291.2]	284.5 [271.8**–**293.0]	270 [242.8**–**292.8]	0.31
*p*‐value by group	0.81	0.62	0.81	0.55	

Abbreviations: ICD, implantable cardioverter defibrillator; IQR, interquartile range; LVEF, Left ventricular ejection fraction; RV, right ventricular.

Correlation analysis showed no significant association between LVEF and TR grade (*R* = −0.16, *p* = 0.15) (Figure S4). Similarly, analysis of the correlation between RV pacing rate and TR grade showed no significant relationship (*R* = −0.11, *p* = 0.41) (Figure S5). Additionally, TR grade did not differ significantly between patients with DDD and VVI pacing modes (*p* = 0.64) (Figure S6).

## 4 DISCUSSION

The worsening TR severity following CIED placement is a known phenomenon, yet the exact pathogenesis is not entirely certain. The prevailing theories include natural progression of disease and lead‐associated mechanical effects.^7^ Our findings suggested that not only is the exacerbation of TR likely lead related, there may be a size‐dependent relationship between the caliber of the lead and worsening TR severity.

Of the CIEDs analyzed, ICDs carry the bulkiest leads, with many of the commercially available devices having a lead body diameter ranging from 2.27 to 2.87 mm.^8^ By contrast, RV pacing leads are around 2 mm in diameter.^9^ His‐PM is a relatively newer configuration to ventricular pacing by way of conduction through the His‐Purkinje system. With a lead body diameter of 1.4 mm, His‐PM leads are not only the thinnest of the devices analyzed but also circumvent crossing the tricuspid valvular apparatus by exploiting the supravalvular position of the proximal‐most extent of the His‐bundle.^10^, ^11^ Additionally, His‐PM preserves ventricular geometry and ensures better ventricular synchronization, which may result in less impact on the progression of tricuspid regurgitation.^12^

We found that ICD placement was significantly associated with worsening TR, and His‐PM placement showed no increase in TR severity. Along this spectrum, RV‐PM leads fell in‐between, as postdevice implantation showed a probable clinically significant increase in TR grade, although statistical significance was not achieved. As such, the direct relationship between lead diameter and TR severity became apparent, as we found that thinner diameter leads resulted in lower rates of postimplantation TR, and CIED leads that are not transvalvular had little to no effect on TR severity.

The most common indication for ICD placement in our patients was dilated cardiomyopathy. Compared to those who underwent PM placement, patients who underwent ICD placement were more likely to exhibit reduced RV function pre‐ and postimplantation and presented with higher rates of preimplant RV dilation, factors which are known to exacerbate functional TR.^13^ At a median follow‐up time of 34 months, we did not necessarily observe worsening right ventricular function in this group. Furthermore, we found no differences in right‐sided pressures following device implantation, supporting the impression that TR is likely related to the presence of CIED leads rather than worsening causes of functional TR.

As we have reported the size‐dependent relationship between lead calibers on TR severity, it is essential to recognize the underlying mechanisms by which this may occur. Transvalvular leads can affect valve leaflet coaptation by direct impingement of the leaflets, adherence to the leaflet at the annular level, or tethering of the chordae at the subannular level.^5^ In this setting, the thicker ICD leads may accelerate fibrosis, and their higher stiffness can worsen leaflet mal‐coaptation.^14^

A study by Breeman et al. assessed the severity of TR in patients who underwent subcutaneous ICD (S‐ICD) placement compared to those with transvenous ICDs (TV‐ICDs).^15^ Their findings were in line with ours and supported that TR was attributed to the presence of transvalvular ICD leads rather than morphologic changes in the right side of the heart. In recent years, leadless pacemakers became commercially available, which allowed for the assessment of the effect of pacemaker leads on TR severity.^16^ A prospective study by Salaun et al. showed that there were no significant changes in TR severity, RV dimension, or right‐sided pressures following leadless pacemaker implantation. However, follow‐up was limited to 2 months, and as of now, long‐term effects of leadless pacemakers on TR have yet to be reported.

### 4.1 Limitations

As with retrospective chart reviews, this study was limited by the information only obtainable through the electronic medical record. Information that may not have been entered in the system but may have otherwise been relevant to our study could not be included. Furthermore, our study population was limited to devices implanted by a single electrophysiologist at a metropolitan tertiary care center; this operator intentionally placed all RV pacing leads on the mid‐septum and avoided apical lead location. Therefore, our findings may not necessarily be generalizable to all device patients, many of whom have RV pacing leads placed at the RV inferior‐apex.

Statistical analysis was constrained by the small sample size, limiting our ability to adjust for multiple confounders without significantly reducing statistical power. Furthermore, comparisons across the three device groups were performed without correction for multiple comparisons, which may increase the likelihood of type I error; thus, findings should be interpreted with caution. Future studies with larger data sets are needed to allow for more comprehensive adjustments and a better understanding of the impact of heart failure and pacing parameters.

## 5 CONCLUSIONS

The effect of CIED leads on TR represents a spectrum related to the type of lead and whether the tricuspid valve is traversed. ICD leads, which are bulkiest and traverse the valve, were associated with significantly worsened TR. His‐PM leads, which do not traverse the valve, had no apparent effect on TR. The impact of RV‐PM leads (placed on the mid RV septum) on TR severity appears in between ICD and His‐PM leads with possible worsening of TR.

## CONFLICT OF INTEREST STATEMENT

The authors declare no conflict of interests for this article.

## ETHICS APPROVAL STATEMENT

This study was approved by the Institutional Review Board (IRB) of Thomas Jefferson University.

## PATIENT CONSENT STATEMENT

N/A.

## PERMISSION TO REPRODUCE MATERIALS

N/A.

## CLINICAL TRIAL REGISTRATION

N/A.

## Supporting information


**Supplementary Figure 1.** Predicted probabilities of TR progression with ICD use, stratified by severity.

**Supplementary Figure 2.** Predicted probabilities of TR progression with RV‐PM use, stratified by severity.

**Supplementary Figure 3.** Predicted probabilities of TR progression with His‐PM use, stratified by severity.

**Supplementary Figure 4.** Correlation of TR grade with LVEF.

**Supplementary Figure 5.** Correlation of TR grade with RV pacing rate.

**Supplementary Figure 6.** Relationship of TR grade with pacing mode of CIEDs.

**Supplementary Table 1.** Ordinal logistic‐regression estimates of TR progression by CIED type.

**Supplementary Table 2.** Two‐sided and directional *p*‐values for pre‐ vs. post‐implant TR severity according to CIED type.

## Data Availability

The data that support the findings of this study are available from the corresponding author, Behzad B. Pavri, upon reasonable request.
