# Supplementary figures and images for: Differing impacts of cardiac implantable electronic device leads on tricuspid regurgitation
Source: J Arrhythm. 2025 Jul 7;41(4):e70133. doi: 10.1002/joa3.70133 (PMC12234372; doi:10.1002/joa3.70133)

## ICD Ordinal Logistic Regression Results

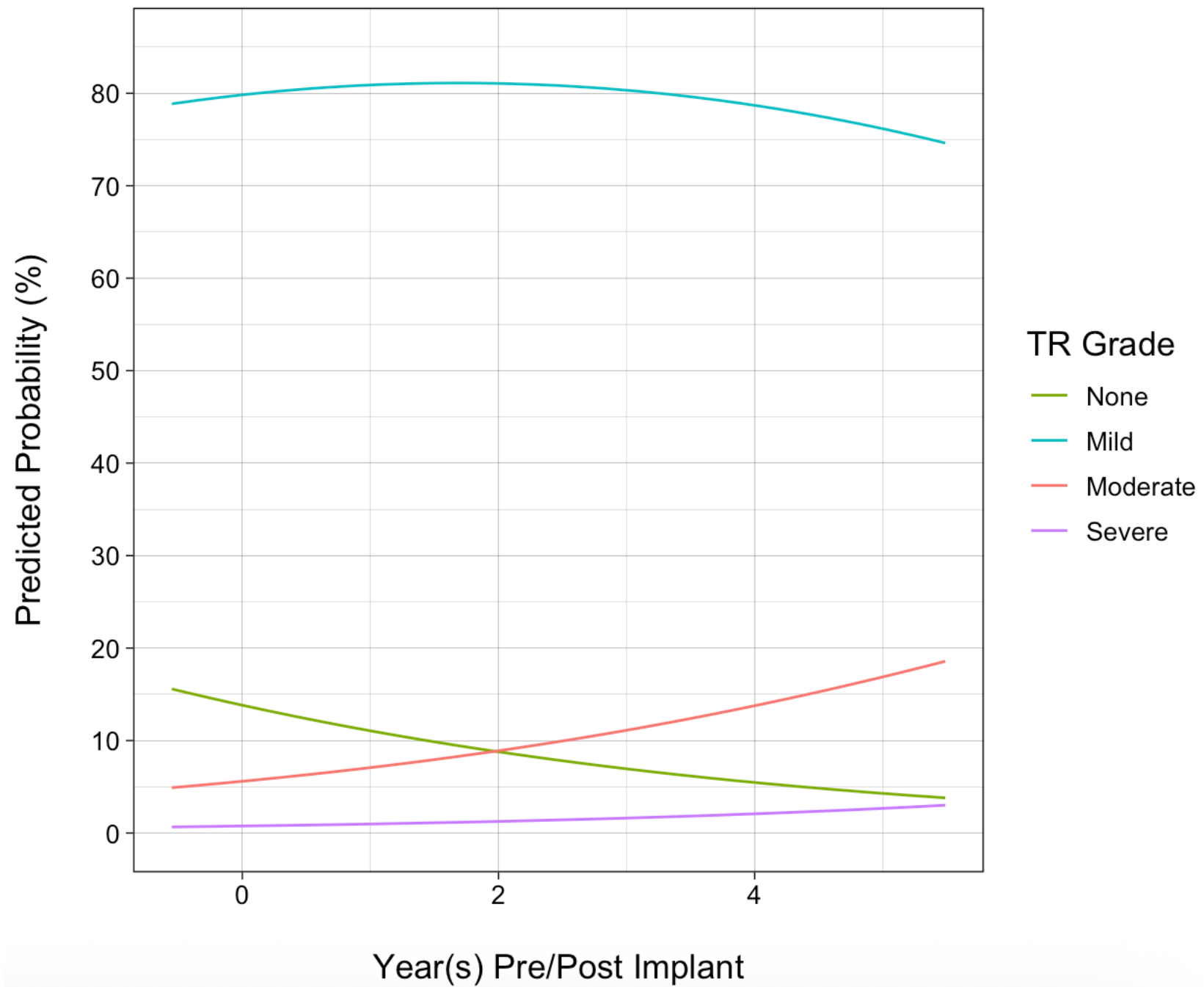

Supplement: Supplementary file 1 — Supplementary Figure 1. Predicted probabilities of TR progression with ICD use, stratified by severity. [file JOA3-41-e70133-s006.pdf]

## RV-PM Ordinal Logistic Regression Results

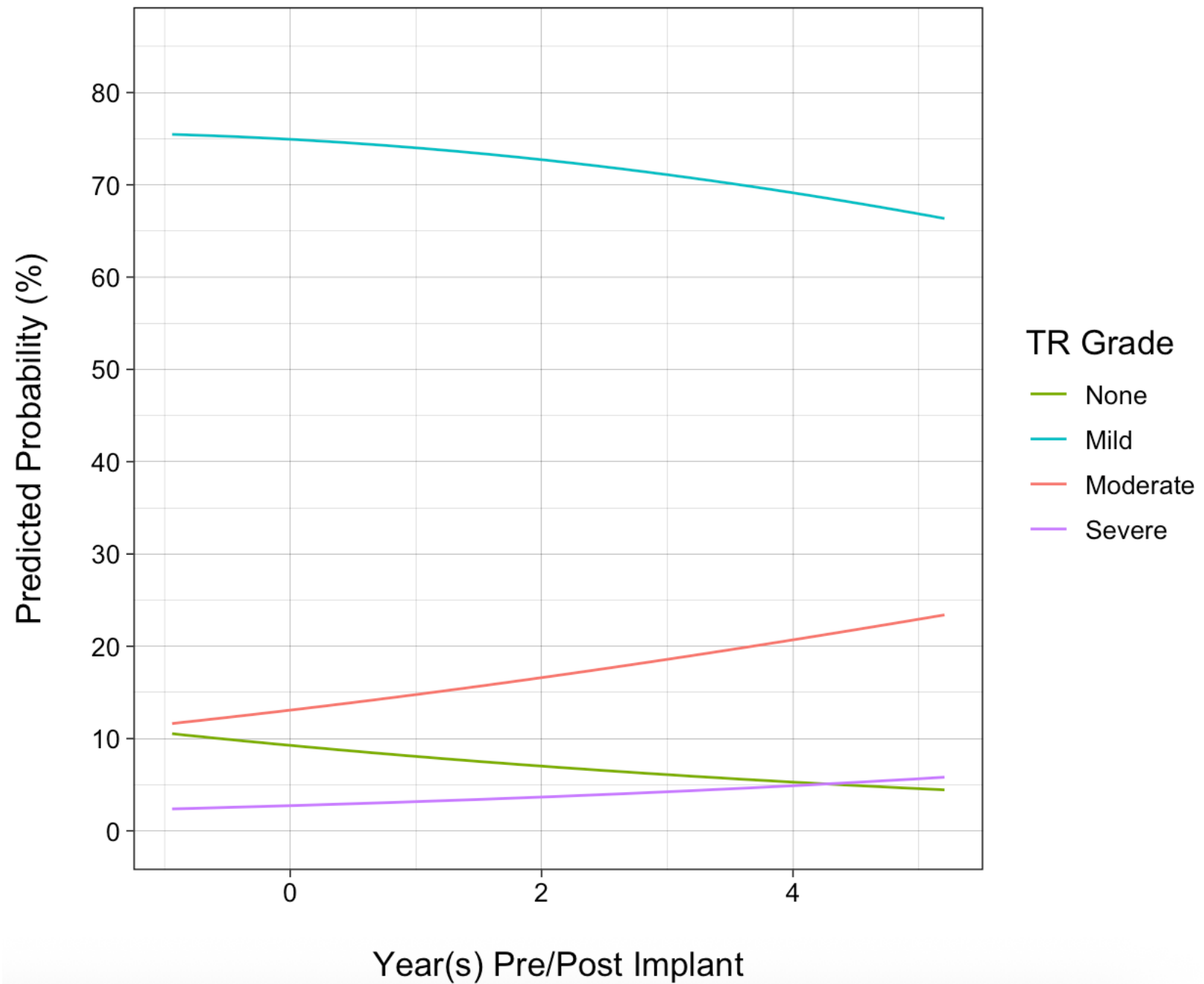

Supplement: Supplementary file 2 — Supplementary Figure 2. Predicted probabilities of TR progression with RV‐PM use, stratified by severity. [file JOA3-41-e70133-s004.pdf]

HIS-PM Ordinal Logistic Regression Results

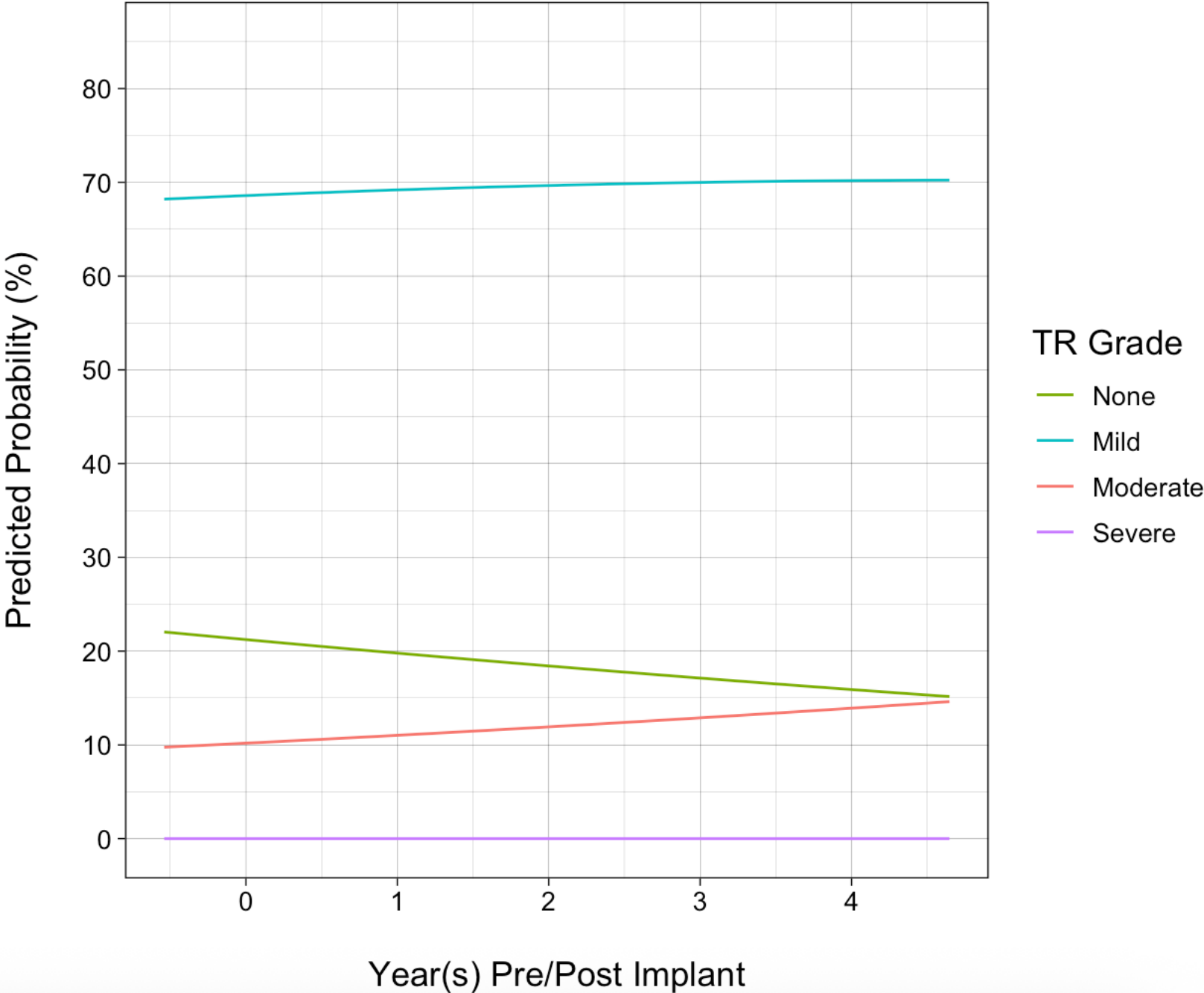

Supplement: Supplementary file 3 — Supplementary Figure 3. Predicted probabilities of TR progression with His‐PM use, stratified by severity. [file JOA3-41-e70133-s008.pdf]

TR Grade

$R = -0.16, p = 0.15$

4  
3  
2  
1  
0

20

40

60

Post-Procedure LVEF %

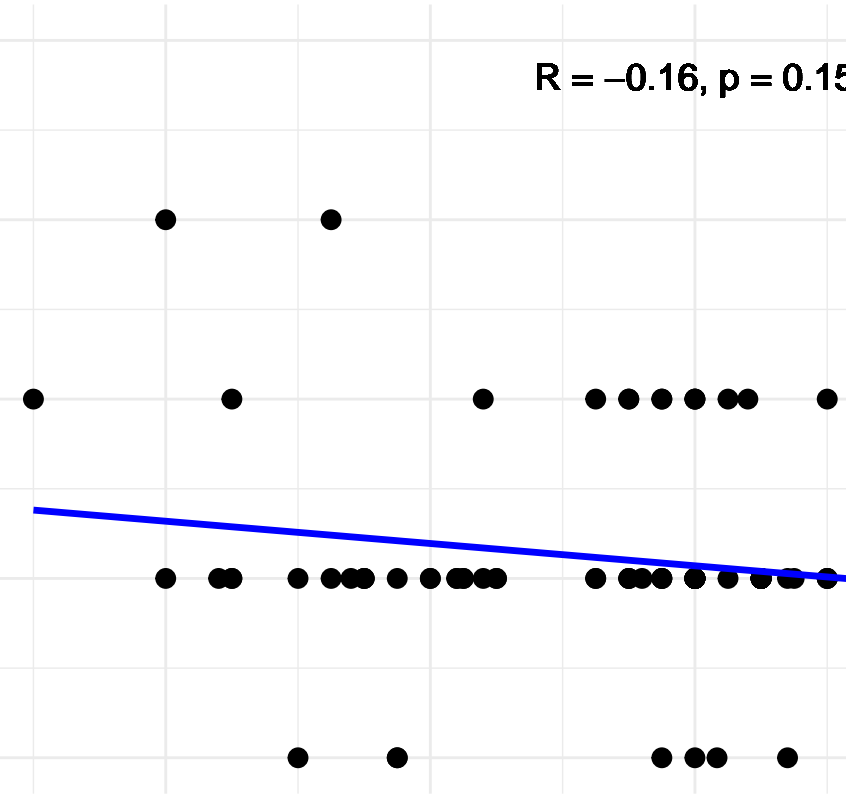

Supplement: Supplementary file 4 — Supplementary Figure 4. Correlation of TR grade with LVEF. [file JOA3-41-e70133-s007.pdf]

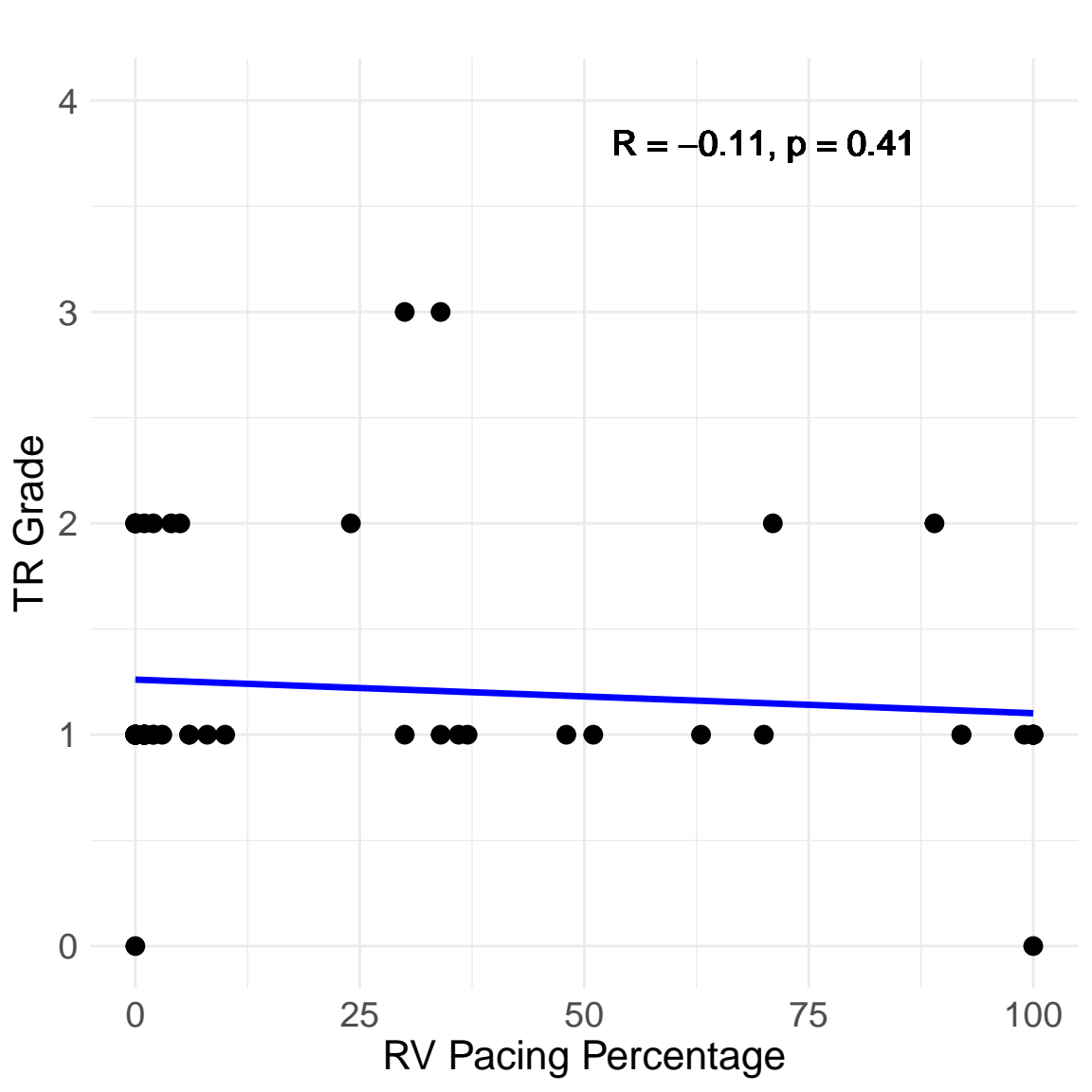

Supplement: Supplementary file 5 — Supplementary Figure 5. Correlation of TR grade with RV pacing rate. [file JOA3-41-e70133-s001.pdf]

TR Grade

3

2

1

0

DDD

VVI

$p = 0.64$

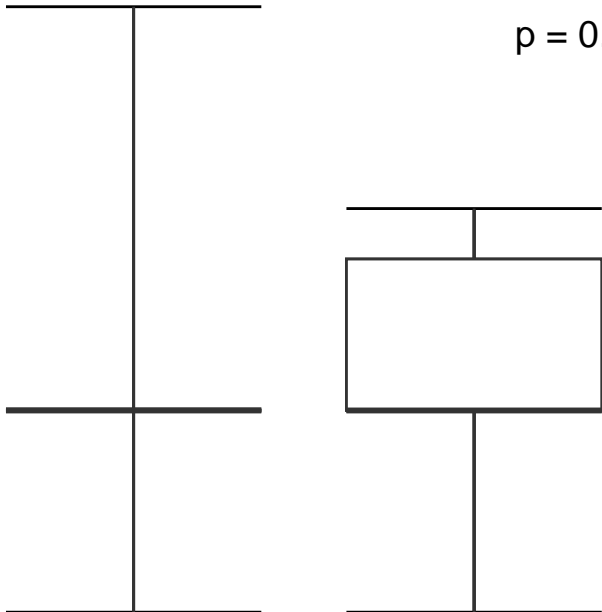

Supplement: Supplementary file 6 — Supplementary Figure 6. Relationship of TR grade with pacing mode of CIEDs. [file JOA3-41-e70133-s003.pdf]
