# Supplementary material for: Differing impacts of cardiac implantable electronic device leads on tricuspid regurgitation
Source: J Arrhythm. 2025 Jul 7;41(4):e70133. doi: 10.1002/joa3.70133 (PMC12234372; doi:10.1002/joa3.70133)
Supplement: Supplementary file 7 — Supplementary Table 1. Ordinal logistic‐regression estimates of TR progression by CIED type. [file JOA3-41-e70133-s005.docx]

**Supplementary Table 1.** Two-sided and directional P-values for Pre- vs Post-Implant TR Severity according to CIED type

|  | ICD | RV | His Bundle |
| --- | --- | --- | --- |
| two-sided | 0.071 | 0.1986 | 0.777 |
| one-sided, pre<post | 0.035 | 0.099 | 0.682 |
| one-sided, pre>post | 0.977 | 0.906 | 0.388 |
