# Supplementary material for: Differing impacts of cardiac implantable electronic device leads on tricuspid regurgitation
Source: J Arrhythm. 2025 Jul 7;41(4):e70133. doi: 10.1002/joa3.70133 (PMC12234372; doi:10.1002/joa3.70133)
Supplement: Supplementary file 8 — Supplementary Table 2. Two‐sided and directional p‐values for pre‐ vs. post‐implant TR severity according to CIED type. [file JOA3-41-e70133-s002.docx]

**Supplementary Table 2.** Ordinal Logistic-Regression Estimates of TR Progression by CIED type

| **Group** | **Odds Ratio (OR)** | **Lower Bound (95% CI)** | **Upper Bound (95% CI)** | **P-Value** |
| --- | --- | --- | --- | --- |
| ICD | 1.29 | 0.91 | 1.85 | 0.158 |
| RV-PM | 1.16 | 0.95 | 1.43 | 0.152 |
| HiS-PM | 1.09 | 0.67 | 1.79 | 0.718 |
